# COVID-19 Stress and Mental Health of Students in Locked-Down Colleges

**DOI:** 10.3390/ijerph18020771

**Published:** 2021-01-18

**Authors:** Xueyan Li, Ping Fu, Changyu Fan, Miao Zhu, Min Li

**Affiliations:** School of Sociology, Central China Normal University, Wuhan 430079, China; lixueyan@mail.ccnu.edu.cn (X.L.); fanchangyu@mail.ccnu.edu.cn (C.F.); zhuzhu@mails.ccnu.edu.cn (M.Z.); lizimin703@mails.ccnu.edu.cn (M.L.)

**Keywords:** COVID-19, university and college student, mental health, post-traumatic stress disorder

## Abstract

The impact of the COVID-19 pandemic on the mental health of students in locked-down colleges remains obscure. This study aimed to explore influencing factors for the psychological impact of COVID-19 on Wuhan college students, post-traumatic stress symptoms in particular, so as to inform evidence-based strategy development to ameliorate such adverse impacts. An online survey was conducted from 26 to 29 April 2020, and 4355 students enrolled in Wuhan universities and colleges participated. Post-Traumatic Stress Disorder via the Impact of Event-Scale-Revised was assessed. COVID-19 disproportionately affected older male Master’s and doctoral students living in Wuhan. The overall prevalence of PTSD was 16.3%. The three-level socio-interpersonal model of PTSD was empirically validated, and college students faced individual level risks such as infection with COVID-19, close relationship level risks such as family support (infection suspicion of family members, the loss of loved ones, and the family income decrease) and online course difficulties (little interaction, disturbing learning environment, and difficulty in adaption), and distant level risks such as excessive collection of personal information, estrangement of family relatives, and harassment and insult from strangers. The findings suggest the severity of the psychological impact of COVID-19. Mental health services reducing PTSD should be provided. Students who have lost loved ones and suffered family financial loss should be given particular care.

## 1. Introduction

The COVID-19 disease has been considered a pandemic since 11 March 2020. As the World Health Organization reported, as of 7 June 2020, there have been 6,799,713 confirmed cases of COVID-19 globally, including 397,388 deaths, and the pandemic continues to spread [1]. Wuhan City was the first city to be ferociously hit and severely affected by the large-scale COVID-19 outbreak. Wuhan City, Hubei Province is home to 83 universities and colleges and 1.2 million students, arguably the largest number of university and college students (college students, thereafter) among all the cities in the world. Meanwhile, Wuhan City, the geographic center of China, known as the “Thoroughfare of Nine Provinces”, is the largest water, land, and air transportation hub, and the financial, commercial, trade, and cultural center of inland China, with a population of more than 11 million people. The large student population in this rapidly moving and closely interactive city is under the vicious spell of the COVID-19 pandemic. Having experienced and witnessed the unpredictable, uncontrollable, and fierce attack of COVID-19 on life and humanity, individuals in severely affected geographical regions such as Wuhan are prone to develop a series of trauma-relevant psychological symptoms, namely Post-Traumatic Stress Disorder (PTSD). PTSD is characterized by intrusion, avoidance, and hyperarousal symptoms, with significant functional impairment or distress [2,3].

Recent studies have examined the psychological impact of the COVID-19 pandemic on medical care workers [4,5,6], children [7,8], older adults [9,10], and the general public [11,12,13,14,15,16,17,18,19,20]. For example, a study [15] reported that the prevalence of post-traumatic stress symptoms (mainly acute stress disorder symptoms) was 7% in residents of Wuhan and the surrounding cities between 30 January and 8 February 2020, one month after the COVID-19 outbreak. Another study [17] conducted in a similar time period (28 January to 5 February 2020) reported a similar prevalence (7.6%) of intrusive and avoidance symptoms in a convenience sample of the general public of Liaoning Province. According to public records, there were 14,982 confirmed cases in Wuhan and 27,100 confirmed cases in Hubei Province on 8 February [21], while there were 89 confirmed cases in Liaoning Province on 5 February [22]. Given that the severity of the pandemic in these two areas was widely different, it would not be surprising to see different traumatic psychological consequences. However, the PTSD prevalence rates reported in both studies were quite similar, and such results were quite puzzling and unexpected. Furthermore, another survey study [23] performed from 19 February to 6 March 2020 found that medical health workers had a higher prevalence of insomnia, anxiety, depression, somatization, and obsessive-compulsive symptoms than nonmedical health workers in China. The disparity in the psychological impact of COVID-19 pandemic on different populations was expected and empirically confirmed. To our knowledge, there has been only one study [24] on the psychological impact of the COVID-19 pandemic on Chinese university students. This study was conducted in Chengdu Province and Chongqing City one month after the COVID-19 outbreak, and it reported a PTSD and depression prevalence of 2.7% and 9.0%, respectively.

Such studies offer a glimpse of the possible psychological impact of the COVID-19 pandemic. So far, most studies have been conducted within one or two months of the COVID-19 outbreak and have mainly focused on its immediate impact. At that time, the number of infections drastically increased daily, the public was in desperate need of clear and definitive information on the virus, and there were severe shortages of medical workers and resources in hospitals and a serious lack of personal protection equipment in markets. All these stressors easily trigger reactions, especially acute stress disorders. The high prevalence of such symptoms in people living in the hardest-hit areas and medical health workers on the frontlines of combating the virus is therefore expected.

Moreover, college students may face different risks during the COVID-19 pandemic. First, the college students who returned to their hometown from Wuhan may experience the fall from being “the pride of the community” to being “a well-known suspected (or later confirmed) virus carrier”. The wide and thorough screening and inspection measures put Wuhan college students in spot, and the unwanted attention was paid along with many negative narratives. Social affects, such as shame, guilt, and anger, emerge from such social interaction. These schematic post-traumatic cognitive changes were an integral part of the core symptoms of PTSD [25,26]. Second, in response to the strict or even aggressive anti-COVID measures adopted all over China, these students had to isolate themselves at home or in a dedicated quarantine facility for at least two weeks and report their temperature and physical status at least twice a day. Isolation, in combination with fear and worry of contagion, anxiety, stigma, and potential misinformation overload, causes stress and is associated with a burden on psychological health. Third, no matter whether students were in Wuhan or elsewhere, they all had to stay at home for an indefinite time period after the two-week isolation. A rapid review [27] reported the negative psychological effects of quarantine, including post-traumatic stress symptoms, confusion, and anger. The stressors include longer quarantine durations, infection fears, frustration, boredom, inadequate supplies, inadequate information, financial loss, and stigma. Furthermore, the policy brief on COVID-19 and mental health issued by the UN Secretary noted that home isolation increases the risk of witnessing or being exposed to domestic violence and abuse [28]. Besides the restraint of normal social life, students had to re-habituate and re-adapt themselves in their “old” home, where their parents may have already been used to the “empty-nest” life since they went to college. Fourth, students who lived in rural areas where the internet infrastructure was inadequate may have had difficulty accessing online learning platforms and resources and became angry, frustrated, helpless, and worried about their academic work. In addition, young people who lacked in-person contact with their peers and teachers may have had difficulty concentrating or were irritable, restless, and neurotic during the long and indefinite period of online learning at home. The lack of in-person social interaction with their peers and teachers and the challenges relevant to the online learning stimulate negative moods.

To sum up, the risks faced by Wuhan college students during the pandemic call for a socio-interpersonal perspective in explaining of the possible psychological impacts. According to the three-level socio-interpersonal model of PTSD proposed by Maercker and Horn [29], the first individual level comprises intrapersonal features or impairments. For these students, the individual level factors include their demographic characteristics such as gender, age, and academic programs, and the factors relevant to trauma severity, such as where they live (in Wuhan or not, in urban or rural areas), isolation types (home or hospital isolation), and their physical status (COVID-19 infected or not). The second level is constituted by their close relationships. For these students, it involves social support from their close family relations, and online interaction with peers and teachers. The third level comprises interaction between the trauma victim and distant social groups, and reflects the social influences on the individual processing the trauma. For these students, the social exclusion and stigma from their community or general public exert effects on their post-trauma reaction.

This study intends to explore influencing factors for the psychological impact of COVID-19 of Wuhan college students, post-traumatic stress symptoms in particular, so as to inform evidence-based strategy development to ameliorate such adverse impacts. Particularly, a three-level socio-interpersonal model of PTSD was empirically examined to explain the PTSD symptoms of students in locked-down colleges during the COVID-19 pandemic.

## 2. Methods

### 2.1. Design, Participants, and Procedure

This study is part of a large-scale survey, COVID-19 Impact Survey of Faculty and Students of Wuhan Universities and Colleges (CFSW), which is a cross-sectional study conducted via an online survey from 26 to 29 April 2020. The study was performed 4 months after the COVID-19 outbreak in Wuhan [30]. As of 26 April, there had been no new confirmed cases of COVID-19 in Hubei Province for 22 consecutive days, and no new confirmed cases in Hubei Province except Wuhan for 52 consecutive days. It had been 18 days since the lockdown of Wuhan City was lifted on 8 April 2020 [31]. Informed consent was obtained from the participants for collecting data.

For non-infected students, a multistage random sampling approach was adopted. All 83 universities and colleges located in Wuhan are affiliated with different departments, i.e., 8 public universities are affiliated with central ministries and commissions of the state, 15 public universities and 29 public junior colleges are affiliated with the province, and 23 private universities and 8 private junior colleges are affiliated with the Hubei Province Department of Education. First, universities and colleges were randomly selected according to their affiliation categories, and the sampled student size in each sampled university and college was proportional to the size of the student population. Second, 30% of the schools and departments of the sampled university and colleges were systematically selected, and the sampled student size in each sampled school and department was proportional to the size of the student population. Third, the size of the student population in each sampled school and department was proportional to the size of the different programs (i.e., junior bachelor, bachelor, master’s, and doctoral programs). Then, the size of the student population was equally distributed among each grade of different programs. For each grade of different programs, students were randomly selected according to their student ID number.

The sampling instructions together with a detailed example were provided to the coordinator, who also acted as an administrative officer of each sampled university and college. A support WeChat group was established to facilitate the sampling process of coordinators. Having finished the sampling in each sampled university and college, one facilitator who also acted as the administrative officer at each sampled school and department was appointed to further randomly select students.

Of all 83 universities and colleges located in Wuhan, 13 were randomly selected, including 2 state-level universities and 11 province-level universities and colleges. Overall, 4285 non-infected students were selected from the sampled 13 universities and colleges. All 70 students who were infected with COVID-19 in participating universities and colleges were included in the survey. The total obtained sample size of students was 4355.

All participants completed the online survey via a professional data collecting platform (http://ringsurvey.com/platform). The online survey included questions on socio-demographic and psychological variables. Participants who had not completed the survey received a warning on unanswered questions from the online platform; however, they were free to stop the survey without receiving a warming from the platform. As a result, the valid participants were those who completed all the questions of the online survey. All participants gave their informed consent for inclusion before they participated in the study. The study was conducted under the Declaration of Helsinki, and the protocol was approved by the Academic Board of Central China Normal University.

### 2.2. Measurements

For the individual level, information of students’ socio-demographic characteristics and COVID-19 trauma severity were collected. Socio-demographic information, such as gender, age, program (i.e., junior bachelor program, bachelor program, Master’s program, or doctoral program), discipline (i.e., liberal arts and humanities or science and engineering), and living place during the pandemic (i.e., mostly living in Wuhan, mostly living in another place in Hubei, or mostly living in other provinces) were collected in the survey. Living in a rural area indicated worse access to internet, which posed a challenge for students’ online learning. The variable of living place during the pandemic reflected the risk of COVID-19 infection since the pandemic broke out first in Wuhan. Variables of COVID-19 trauma severity included students’ isolation type and physical status. Students were asked the following questions: “What was your isolation status after returning from Wuhan?” (1) Home isolation, (2) centralized isolation (hotel or designated places), and (3) hospital isolation treatment; “During the pandemic, did you have the following symptoms” (Multiple-option question)? (1) Itchy throat and dry cough, (2) weakness and joint pain, (3) fever, (4) confirmed influenza, (5) pneumonia, but not confirmed COVID-19 infection, and (6) confirmed COVID-19 infection. Those who chose option (6) “confirmed COVID-19 infection” were re-coded as infection with COVID-19 (value 1), and the rest as no-infection (value 0).

For the close relationship level, information about social support and online learning-related interaction was collected. Students were asked the following questions: “During the pandemic, did your family members experience any of the following conditions?” (1) Family members were diagnosed/infected with COVID-19 (yes or no); and (2) family members were suspected with COVID-19 (yes or no). “Have you lost a loved one to COVID-19?” (1) Yes, immediate family members, (2) yes, other relatives, (3) yes, friends, colleagues, or classmates, (4) yes, neighbors, and (5) No. This variable was re-coded into two values, i.e., No = 0, and others = 1. “How is your family income during the pandemic?” (1) Increased, (2) decreased by above 50%, (3) decreased by 30–50%, (4) decreased by under 30%, and (5) basically no change. This variable was re-coded into two values, i.e., increased and basically no change = 0, and others = 1.

Two questions reflected students’ online learning-related interaction and difficulties. “Currently, do you take online courses?” (1) Yes, and (2) no. “What were the difficulties you faced with online courses? (multiple choice)” (1) Little interaction and poor learning effect, (2) disturbed learning, (3) slow internet access speed at home, (4) difficulty in adapting to online courses, and (5) others.

For the distant social level, social exclusion and stigma encountered by students were measured. “Have you encountered the following situations because of your school experience in Wuhan?” (1) Personal information was excessively collected; (2) identity information was maliciously spread; (3) intentional estrangement of family relatives; (4) intentional alienation of neighbors and friends; (5) discrimination/exclusion of residents in the community; (6) harassment, abuse, or insult; and (7) other unpleasant experiences.

For PTSD, the question was: “Since April 8, how is your mental state consistent with the following statements?” PTSD was assessed via the Chinese version of the Impact of Event-Scale-Revised (IES-R) [32,33], a 25-item self-reported scale assessing the severity of post-traumatic disorder symptoms due to traumatic events such as the COVID-19 pandemic. Three dimensions, including intrusion, avoidance, and hyperarousal symptoms, were assessed. Participants were asked to rate all the items using a 5-point scale (0 = never, 1 = rarely, 2 = sometimes, 3 = often, 4 = always). The word “event” was replaced with “COVID-19 pandemic” in the items. The Cronbach coefficient was 0.89, 0.82, and 0.86, respectively, for the subscales of Intrusion, Avoidance, and Hyperarousal, and 0.92 for the whole scale of IES-R. Three items (2 from the Avoidance and 1 from the Hyperarousal subscales) were removed from further analysis because the corresponding Cronbach coefficient of the subscale was higher if removed.

For PTSD screening, a recommended cutoff of ≥1.5 was used for the average of each subscale score and the whole scale score for the following analysis. This cutoff value was established against the PTSD Checklist (PCL) in a community sample, with an overall diagnostic power of 0.88, a sensitivity of 0.91, a specificity of 0.82, a positive predictive power of 0.90, and a negative predictive power of 0.84 [34].

### 2.3. Statistical Analyses

χ^2^ tests were used to compare differences in categorical variables between groups. Multivariate regression analyses were performed on the total score of PTSD. Data were analyzed using SPSS 21.0.

## 3. Results

### 3.1. Prevalence and Socio-Demographic Characteristics

In total, 4355 participants from 13 universities and colleges in Wuhan completed the survey in this study. Table 1 presents the socio-demographic features of the whole sample and compares the 4285 non-infected students with infected 70 students.

Overall, 708 of 4355 participants (16.3%) met the criteria for PTSD symptoms. The prevalence of PTSD among infected students was significantly higher than that among non-infected students (27.1% vs. 16.1%, respectively, *p* < 0.05). Their average score on each item was higher than the cutoff value of 1.5.

The comparison between infected and non-infected students regarding socio-demographic information and PTSD is shown in Table 1. The infected students demonstrated different socio-demographic characteristics compared to non-infected students. Compared to non-infected students, infected students were disproportionately male (67.1 vs. 50.5%, *p* < 0.01), older (68.6 vs. 43.7% born before 2000, *p* < 0.001), in Master’s and doctoral programs (41.5 vs. 12.3%, *p* < 0.001), in state-affiliated universities (57.1 vs. 24.3%, *p* < 0.001), and living in Wuhan (45.7 vs. 11.6%, *p* < 0.001) during the COVID-19 pandemic. These students disproportionately lived in an urban area (74.3 vs. 37.5%, *p* < 0.001) of China. The infected students showed higher prevalence rates of Intrusion symptoms (37.1 vs. 25.8%, *p* < 0.05), and higher IES-R total scores (27.1 vs. 16.1%, *p* < 0.05).

### 3.2. Factors Related to the Occurrence of PTSD

The results of multivariate regression analyses are presented in Table 2. Based on the three-level socio-interpersonal model of PTSD, three models were constructed and examined on the basis of the baseline model. In the baseline model, compared to the bachelor students, master’s students faced significantly higher risk of PTSD (β = 0.049, *p* = 0.002). In the 1-level (i.e., individual level) model, two factors including whether students lived in Wuhan during the pandemic and whether they were infected with COVID-19, were tested. The results showed that the students who were infected with COVID-19 faced a significantly higher risk of PTSD (β = 0.052, *p* = 0.001), compared with those who were not. Students were asked their isolation status after returning from Wuhan. Since all students living in Wuhan during the pandemic did not answer this question and did not provide information on their isolation status due to the item measurement fault, the variable of isolation status was not included in the model. However, a separate ANOVA of isolation status on PTSD showed no significant difference among three statuses of isolation on PTSD.

When the factors of close relationship level were included into the model, the predicting effect of whether students were infected with COVID-19 was no longer significant. On the close relationship level, whether students’ family members were suspected with COVID-19 (β = 0.047, *p* = 0.014), whether they had lost a loved one to COVID-19 (β = 0.051, *p* = 0.002), and whether their family income decreased (β = 0.068, *p* < 0.001) significantly predicted their PTSD symptoms. Due to the lockdown of universities and colleges, 3752 out of 4355 students (86.2%) had to take online courses during the pandemic. The results showed that, for these students, the online course difficulties including the lack of teacher-student and student-student interaction (β = 0.093, *p* < 0.001), the disturbed learning environment (β = 0.074, *p* < 0.001), and the difficulties in adapting oneself to the online courses (β = 0.094, *p* < 0.001), were significant predictors of PTSD symptoms.

In the three-level model, the distant social impacts were taken into consideration. The results showed that, during the pandemic, the widely existence of excessively collecting personal information (β = 0.072, *p* < 0.001), the family relatives’ intentional estrangement (β = 0.051, *p* = 0.010), and the perceived discrimination and exclusion from community and village (β = 0.053, *p* = 0.002) posed significant negative effects on students’ mental health.

## 4. Discussion

### 4.1. COVID-19 Disease Disproportionately Affects Older Male Students with a Higher Education Level Who Lived in Wuhan during the Pandemic

This study found that, socio-demographically, COVID-19 disease disproportionately affected older male Master’s and doctoral students who lived in Wuhan during the pandemic. There are 1.2 million university and college students in Wuhan. According to public website records, most universities and colleges ended autumn-semester class and started their winter holidays around 11–13 January 2020. Among the sampled 13 universities and colleges, two universities with the largest number of infected students started their winter holidays on 11 and 12 January. Around this time, when the COVID-19 disease had just emerged [35] and had not become widely spread, most students left for their hometowns. This timeline saved most students who went back to their hometowns outside Wuhan from the vicious attack of COVID-19.

However, students who were in Master’s or doctoral programs usually remained in Wuhan longer due to unfinished research projects and laboratory experiments and thus, unfortunately, had a higher risk of being caught by the COVID-19 pandemic. Generally speaking, Master’s and doctoral students are older than junior bachelor and bachelor students. Therefore, students’ ages are highly correlated with their education level, which explains why COVID-19 disease disproportionately affects older Master’s and doctoral students living in Wuhan during the pandemic. The finding that male students were more likely to be infected with COVID-19 than females is consistent with the gender differences found in the general public [36,37], and some researchers proposed an immunity explanation for such gender differences [36].

### 4.2. The Prevalence of PTSD Symptoms in College Students is High

This study found that the prevalence of PTSD in students of Wuhan universities and colleges four months after the COVID-19 pandemic was 16.3%. A study of university students of Chengdu Province and Chongqing City between 30 January and 8 February 2020, reported a PTSD and depression prevalence of 2.7% and 9.0%, respectively, one month after the COVID-19 outbreak [24]. Another study of the general public in Wuhan and the surrounding cities between 30 January and 8 February 2020 reported an acute stress disorder symptom prevalence of 7%, which was one month after the COVID-19 outbreak, and the authors predicted that the prevalence may have been more severe before the COVID-19 pandemic was contained and the quarantine was lifted [38]. Researchers found that the depression symptom prevalence of the general public in Taiwan was 3.7% after the SARS epidemic [39].

Compared with these studies, the COVID-19 pandemic appears to have had a greater psychological impact than the SARS epidemic, and such an impact was even greater in the college student population than in the general public of Wuhan over a relatively longer period of time. The significantly higher PTSD, stress, and anxiety of students compared to those employed was also found in another study [19]. This high PTSD prevalence may reflect the multiple and accumulated stressors students faced on individual, close relationship, and distant social levels.

Some researchers [40,41] have argued that PTSD may decrease over time, and only a small proportion of people develop chronic disorders if medically unattended [42]. On the other side, some research stated that the severity of the initial reaction to stress may be predictive of subsequent development and maintenance of PTSD [43]. Thus, if the PTSD symptoms last for longer than 3 months, the risk of developing into chronic PTSD will increase. Such an argument may indicate that now is the time for those students with PTSD to heal and recover to avoid the further development of chronic disorders.

In addition, this study analyzed the differences of PTSD prevalence in two groups, infected and non-infected students, and found that the prevalence in the former group was significantly higher than in the latter group (27.1% vs. 16.1%, respectively). This finding provides direct evidence for the COVID-19 pandemic as a stressor. We have reason to believe that it may be one of the most influential stressors and ignites a series of other stressors, as listed before. Recent studies have consistently found a negative psychological impact of the COVID-19 pandemic on different populations and the general public, but there is little research on the more severe suffering of those diagnosed with COVID-19 disease. The present study provides a glimpse of such suffering with higher rates of PTSD.

### 4.3. Infected Students Have Higher Prevalence of Intrusion, Avoidance, and Hyperarousal Symptoms than Non-Infected Students

This study found that college students had a high prevalence of PTSD, including intrusion, avoidance, and hyperarousal symptoms. In particular, infected students had a significantly higher prevalence of intrusion and PTSD symptoms. Extensive literature [2] provides explanations as to why those who suffered from severe disease are more vulnerable to PTSD. Although COVID-19 is characterized by high infectivity and low mortality, those who contracted the virus at the onset of the pandemic faced medical care resource depletion, uncertain prognosis after a long time of suffering, the fear of infecting others, and unwanted stigmatization and exclusion. All these factors may act as triggers of PTSD symptoms for the infected students.

### 4.4. Risk Factors of PTSD

Given the relatively small number of infected students, this study explored the risk factors of PTSD for all students, not specifically for the infected sub-sample. As proposed in the socio-interpersonal model of PTSD, the risk factors were empirically examined on three levels. On the individual level, the exposure risk was being infected with COVID-19, which was a direct impact of the COVID-19 pandemic on individuals. However, to be noted, such effects seemingly diminished when the factors on the close relationship and distant social levels were involved, and therefore may indicate the greater influence of the latter factors. Another risk factor, i.e., the living places during the pandemic, turned out to be an insignificant factor for PTSD symptoms. In other words, there is no significant difference on PTSD symptoms for students who lived in or outside of Wuhan during the pandemic. This finding may be explained by “the psychological typhoon eye effect”, i.e., people in the epicenter perceive lower risk than others. Such an effect was found in the risk perception research of SARS [44]. Such an effect should be further examined in future research.

On the close relationship level, the factors of family members suspected with COVID-19 or died of COVID-19 significantly predicted students’ PTSD symptoms. The lack of social support and the breakdown of social support structures due to the loss of loved ones were strong predictors of PTSD. Such results were in line with several meta-analyses of the influence of social support on PTSD [45,46]. The anxiety triggered by the suspicion of family members’ COVID-19 infection, especially the uncertainty whether students brought the COVID-19 from Wuhan to their family, posed a significant pressure on students themselves. For students who lost family members to the COVID-19 pandemic, their suffering was severe. Burial and memorial services that provide opportunities for people to say goodbye and have closure with the lost loved one could not be held as usual. This unnatural process of grieving could deeply traumatize people and leave an unhealed psychological scar for a long time. Furthermore, the decrease of family income was a significant risk for PTSD. The inevitable financial loss, mainly the inability to work due to the isolation measures or losing a loved one who had been the family income-earner, put the family in a vulnerable place and stimulated more negative emotional experience.

College students got used to the stable and undisturbed learning environment where teacher–student and student–student interaction were routine. However, as a substitute of normal study life on campus, students had to take online courses at home, which was usually an easily disturbed environment since all family members were home isolated together. Besides, interaction among teachers and students in campus was integral for the social support structure for students. The insufficient interaction meant the decline of social support. The negative impact of online learning difficulties on students’ mental health was empirically supported in this study.

The influence of factors on distant social level, including the excessively collection of personal information, the estrangement of relatives, and the harassment and insults from strangers, was empirically validated in this study. To contain the spread of COVID-19, the Chinese government at all levels widely adopted strict measures, including isolating and monitoring people who traveled from Wuhan to every place around China. The repeated collecting of personal demographic, physical, and other relevant information was a routine during the pandemic, which had a negative impact on students’ mental health. Moreover, the transparency established in the process made students who studied in Wuhan more well-known than ever. The spotlight shed on them further fueled the estrangement of their relatives, and hostility from strangers in the community, even in more distant social circles.

In addition, in all four models, compared with bachelor students, masters’ students faced a significantly higher risk of PTSD. This result was consistent with the prevalence of COVID-19 infection. Master’ students usually got involved in more research activities, and therefore left Wuhan later than bachelor students who usually went back home immediately after finishing their final exams, and therefore masters’ students had a higher risk of being caught by COVID-19.

There are limitations of the present study to bear in mind. The IES-R score in this study did not intend to provide a clinical diagnosis, but rather to identify those with significant traumatic symptoms who require a further evaluation to determine if they meet the full criteria for a diagnosis of PTSD. The data was collected online, and no on-spot support was able to provide for students to answer the survey.

## 5. Conclusions

To conclude, the COVID-19 disease disproportionately attacked older male students in Master’s and doctoral programs who lived in Wuhan during the pandemic. The prevalence of PTSD in university and college students in Wuhan was 16.3%. The COVID-19-infected students had a higher prevalence of intrusion, avoidance, and hyperarousal symptoms than non-infected students. The three-level socio-interpersonal model of PTSD was empirically validated, and students in Wuhan universities and colleges faced individual level risks such as infection with COVID-19, close relationship level risks such as family support (infection suspicion of family members, the loss of loved ones, and the family income decrease) and online learning difficulties (little interaction with teachers and peers, disturbing learning environment, and difficulty in adapting online learning), and distant level risks such as excessive collection of personal information, estrangement of family relatives, and harassment and insults from strangers.

The findings suggest the severity of psychological impact of COVID-19. Mental health services which integrate individual, close-relationship, and distant social factors to reduce PTSD should be provided. Students who lost loved ones to COVID-19 or suffered family income decrease should be given particular care.

## Figures and Tables

**Table 1:** Socio-demographic characteristics of Wuhan college students.

Characteristics	Total (*n* = 4355) %(*n*)	Non-Infected Students (*n* = 4285) %(*n*)	Infected Students (*n* = 70) %(*n*)	χ^2^ Test(*p*)
Gender				7.674(0.006)
Male	50.7(2209)	50.5(2162)	67.1(47)	
Female	49.3(2146)	49.5(2123)	32.9(23)	
Age cohort				47.943(<0.001)
Born before 1995	4.4(193)	4.2(179)	20.0(14)	
Born after 95, but before 2000	39.7(1728)	39.5(1694)	48.6(34)	
Born after 2000	55.9(2434)	56.3(2412)	31.4(22)	
University category				45.255(<0.001)
Public University affiliated with the state	24.8(1082)	24.3(1042)	57.1(40)	
Public University affiliated with the province	30.8(1340)	31.1(1334)	8.6(6)	
Public College affiliated with the province	34.1(1483)	34.1(1461)	31.4(22)	
Private University and college affiliated with the province	10.3(450)	10.5(448)	2.9(2)	
Program				54.402(<0.001)
Junior bachelor	38.6(1683)	38.7(1660)	32.9(23)	
Bachelor	48.6(2118)	49.0(2100)	25.7(18)	
Master’s	10.0(437)	9.7(414)	32.9(23)	
Doctoral	2.7(117)	2.6(111)	8.6(6)	
Discipline				0.727(0.394)
Liberal arts	45.0(1961)	45.1(1933)	40.0(28)	
Science and engineering	55.0(2394)	54.9(2352)	60.0(42)	
Years of current education				4.301(0.231)
1 year	49.4(2152)	49.6(2126)	37.1(26)	
2 years	27.5(1199)	27.4(1175)	34.3(24)	
3 years	15.2(664)	15.2(651)	18.6(13)	
4 years and above	7.8(340)	7.8(333)	10.0(7)	
Living place category				39.730(<0.001)
Rural	39.4(1716)	39.8(1706)	14.3(10)	
Town	22.5(980)	22.7(972)	11.4(8)	
Urban	38.1(1659)	37.5(1607)	74.3(52)	
Living place during pandemic				
Wuhan	12.2(530)	11.6(498)	45.7(32)	75.783(<0.001)
Other places in Hubei	47.7(2079)	48.1(2062)	24.3(17)	
Other provinces	40.1(1746)	40.3(1725)	30.0(21)	
IES-R-Intrusion				4.641(0.031)
No	74.1(3225)	74.2(3181)	62.9(44)	
Yes	25.9(1130)	25.8(1104)	37.1(26)	
IES-R-Avoidance				2.406(0.121)
No	87.5(3811)	87.6(3754)	81.4(57)	
Yes	12.5(544)	12.4(531)	18.6(13)	
IES-R-Hyperarousal				2.985(0.084)
No	84.5(3682)	84.7(3628)	77.1(54)	
Yes	15.5(673)	15.3(657)	22.9(16)	
IES-R-Total				6.192(0.013)
No	83.7(3647)	83.9(3596)	72.9(51)	
Yes	16.3(708)	16.1(689)	27.1(19)	

**Table 2:** Predictors of PTSD symptoms from a socio-interpersonal perspective.

Variables	Baseline Model (*n* = 4355)	1-Level Model (*n* = 4355)	2-Level Model (*n* = 3752)	3-Level Model (*n* = 3752)
**Socio-demographic characteristics**				
Gender (male = 0)	0.012	0.014	0.028	0.043 *
Programs (bachelor = 0)				
Junior bachelor	0.010	0.008	0.004	0.016
Master’s	0.049 **	0.043 **	0.060 ***	0.045 **
Doctoral	0.027	0.024	0.032 *	0.021
Discipline (liberal arts = 0)	−0.021	−0.021	−0.016	−0.013
**Individual level**				
Living place during pandemic (outside Wuhan = 0)		0.012	0.006	0.021
Infected with COVID-19 (no = 0)		0.052 ***	0.023	0.006
**Close relationship level**				
Family infected with COVID-19 (no = 0)			0.003	−0.005
Family suspected with COVID-19 (no = 0)			0.047 *	0.052 **
A loved one lost to COVID-19 (no = 0)			0.051 **	0.055 ***
Family income (increase/no change = 0)			0.068 ***	0.064 ***
**Online course difficulty**				
Little interaction (no = 0)			0.093 ***	0.084 ***
Disturbed learning (no = 0)			0.074 ***	0.057 ***
Slow access speed (no = 0)			0.025	0.021
Difficulty in adaption (no = 0)			0.094 ***	0.090 ***
**Distant social level**				
Excessively collecting personal information (no = 0)				0.072 ***
Maliciously spreading identity information (no = 0)				0.017
Family relatives’ Intentional estrangement (no = 0)				0.051 **
Neighbors and friends intentional alienation (no = 0)				0.020
Discrimination/exclusion (no = 0)				0.035
Harassment, abuse or insult (no = 0)				0.053 **

Note: * *p* < 0.05, ** *p* < 0.01, *** *p* < 0.001.

## Data Availability

The data presented in this study are available on request from the corresponding author. The data are not publicly available due to privacy restrictions.
